# Investigation of hub genes and immune status in heart transplant rejection using endomyocardial biopsies

**DOI:** 10.1111/jcmm.16127

**Published:** 2020-11-23

**Authors:** Meng‐xi Xiu, Yuan‐meng Liu, Wen‐jun Wang

**Affiliations:** ^1^ Medical School of Nanchang University Nanchang China; ^2^ Department of Cardiovascular Surgery The First Affiliated Hospital of Nanchang University Nanchang China

**Keywords:** bioinformatic analysis, differentially expressed genes, heart transplant rejection, immune cell infiltration, protein‐protein interaction network

## Abstract

T cell‒mediated rejection (TCMR) and antibody‐mediated rejection (ABMR) are severe post‐transplantation complications for heart transplantation (HTx), whose molecular and immunological pathogenesis remains unclear. In the present study, the mRNA microarray data set GSE124897 containing 645 stable, 52 TCMR and 144 ABMR endomyocardial biopsies was obtained to screen for differentially expressed genes (DEGs) between rejected and stable HTx samples and to investigate immune cell infiltration. Functional enrichment analyses indicated roles of the DEGs primarily in immune‐related mechanisms. Protein‐protein interaction networks were then constructed, and *ICAM1*, *CD44*, *HLA‐A* and *HLA‐B* were identified as hub genes using the maximal clique centrality method. Immune cell infiltration analysis revealed differences in adaptive and innate immune cell populations between TCMR, ABMR and stable HTx samples. Additionally, hub gene expression levels significantly correlated with the degree and composition of immune cell infiltration in HTx rejection samples. Furthermore, drug‐gene interactions were constructed, and 12 FDA‐approved drugs were predicted to target hub genes. Finally, an external GSE2596 data set was used to validate the expression of the hub genes, and ROC curves indicated all four hub genes had promising diagnostic value for HTx rejection. This study provides a comprehensive perspective of molecular and immunological regulatory mechanisms underlying HTx rejection.

## 1 INTRODUCTION

Over the last 5 decades, heart transplantation (HTx) has become the definitive gold standard surgical approach for patients with end‐stage heart disease, such as heart failure.^1^, ^2^ However, even with immunosuppressive treatments, allograft rejection remains a major cause of morbidity and mortality because the pathogenesis, diagnosis and management of rejection remain highly undefined.^3^, ^4^ According to the International Society for Heart and Lung Transplantation (ISHLT) guidelines, HTx rejection can be divided into T cell‒mediated rejection (TCMR) and antibody‐mediated rejection (ABMR), the diagnoses of which are based on the histology of the endomyocardial biopsies (EMBs).^5^, ^6^, ^7^, ^8^, ^9^

Recently, molecular examination of EMBs has been proposed to improve the precision and accuracy of HTx rejection diagnosis. A new diagnostic system, the Molecular Microscope™ Diagnostic System (MMDx), assesses EMBs in terms of their molecular phenotype, including stable (normal), TCMR, ABMR and injury; the microarray data are available in GSE124897.^10^, ^11^ In the present study, we use bioinformatics analysis to screen the differences in gene expression between TCMR/ABMR and stable HTx samples and identify hub genes. Additionally, abnormal immune cell infiltration in TCMR/ABMR samples and the significant relationship between hub gene expression and immune cell populations, further implicates these hub genes in HTx immunity. Finally, the diagnostic value of hub gene expression in detecting HTx rejection was validated using the GSE2596 data set.

High‐throughput microarray technologies have been increasingly used for the comprehensive identification of potential therapeutic target genes or biomarkers in several kinds of heart diseases.^12^, ^13^, ^14^ However, few studies have investigated the molecular and immunological regulatory mechanisms of HTx rejection by performing bioinformatics analysis. Therefore, the aim of the present study was to identify candidate hub genes and investigate the immune status in HTx rejection through bioinformatics analysis, which deepened our understanding of the diagnosis and treatment of HTx rejection.

## 2 MATERIALS AND METHODS

### 2.1 Data collection and identification of differentially expressed genes (DEGs)

The mRNA microarray data set GSE124897 deposited by Chang et al was downloaded from the GEO database (http://www.ncbi.nlm.nih.gov/geo/), which contained EMBs from HTx recipients, including 645 stable, 52 TCMR and 144 ABMR samples (NCT02670408, an INTERHEART study). The inclusion criteria for selecting the microarray data set were set as follows: (a) the samples detected are heart transplant tissues from homo sapiens, (b) tissues are diagnosed with rejected and stable heart transplant tissues, (c) gene expression profiling of mRNA, and (d) the sample size is greater than 200. The available information on clinical patient/biopsy characteristics is obtained from the results of NCT02670408 (Table 1). The GPL15207 [PrimeView] Affymetrix Human Gene Expression Array was utilized to obtain gene expression profiles. DEGs between TCMR, ABMR and stable HTx samples were screened using the *R 3.5.0* software and the LIMMA package. Adjusted *P* values <.05 and |log_2_ fold change (FC)|> 1 were set as cut‐off standards and indicate statistical significance.^15^

**Table 1:** Characteristics of patients and biopsies

Biopsy characteristics	Biopsies (n = 889)
Days to biopsy post‐transplant	
Mean	787
Median (range)	232 (6‐10,150)
Days to most recent follow‐up after biopsy	
Mean	756
Median (range)	385 (1‐3,854)
Indication for biopsy	
Clinical	154
Follow‐up	108
Protocol biopsy	613
Not recorded	14
Molecular diagnoses	
TCMR	52
ABMR	144
Injury	48
No rejection	645

Patient characteristics	Patients (n = 454)
Mean patient age at first biopsy (range)	51 (2‐78)
Mean donor age (range)	41 (6‐68)
Patient sex	
Male	321
Female	133
Donor sex	
Male	286
Female	145
Not available	23
Patient had a previous failed heart transplant	13
Heart status at last follow‐up	
Alive at last follow‐up	394
Deceased	56
Failed and retransplanted	3
Not available	1
Primary disease ^a^	
Dilated cardiomyopathy	229
Hypertrophic cardiomyopathy	28
Restrictive cardiomyopathy	11
Other cardiomyopathy	37
Congenital heart defect	29
Coronary artery disease	85
Other	34
Not recorded	2

Note

Data are obtained from the results of NCT02670408. Reproduced from Halloran et al, with the permission of American Society for Clinical Investigation.^11^

^a^ Some patients received more than one primary diagnosis.

### 2.2 Functional enrichment analyses of DEGs

Functional enrichment analyses of DEGs were performed using *Metascape*, a powerful tool that can annotate large numbers of genes and perform enrichment analysis.^16^ This tool integrates several authoritative functional databases such as Gene Ontology (GO), Kyoto Encyclopedia of Genes and Genomes (KEGG), and Reactome.

### 2.3 Protein‐protein interaction (PPI) network construction and analysis

Search Tool for the Retrieval of Interacting Genes/Proteins (STRING 11.0; http://string.embl.de/) was used to trace and predict the protein‐protein interaction (PPI) network after the DEGs were imported into the database, with a combined score >0.4.^17^ An open‐source visualization software *Cytoscape 3.6.1* was used to construct and visualize the PPI network. Subsequently, the Molecular Complex Detection (MCODE) plug‐in was used to screen the most significant modules in the PPI network, with node score cut‐off = 0.2, K‐Core = 2, max depth = 100, and degree cut‐off = 4 set as the selection criteria. Furthermore, the cytoHubba plug‐in was utilized with the maximal clique centrality (MCC) method to identify hub genes in the PPI network.

### 2.4 Immune cell infiltration evaluation and analysis

CIBERSORT is an analytical tool used to estimate the relative proportion of 22 human immune cells using gene expression data.^18^ We uploaded the normalized gene expression profiles to the CIBERSORT web portal and set the algorithm to 100 permutations. Samples that met the CIBERSORT *P* < .05 requirements were considered eligible for continued analysis. In each sample, the combined proportions of all immunocyte types were set to equal 1.

### 2.5 Prediction of drug‐gene interactions

Drugs approved by the Food and Drug Administration (FDA) were selected through the Drug‐Gene Interaction Database (DGIdb; http://www.dgidb.org/search_interactions) based on the hub genes identified as promising targets. Drug‐gene interactions were constructed and visualized using *Cytoscape*.

### 2.6 Validation of expression levels and diagnostic value of hub genes

The GSE2596 data set, which includes 27 stable and 16 rejection HTx recipients, was used as a verification data set to evaluate the expression levels of the identified hub genes. Furthermore, receiver operating characteristic (ROC) analysis was used to assess the performance of hub genes in this research using *GraphPad Prism 7.0* software. The area under the ROC curve (AUC) was then computed.

### 2.7 Statistical analysis

*GraphPad Prism 7.0* software was used for statistical analysis: (a) Two‐tailed Student's *t* test was used to analyse the differences between immune cell populations in eligible rejection and stable HTx samples. (b) ROC curve analysis was used to determine the diagnostic effectiveness of hub genes in the verification GSE2596 data set.

## 3 RESULTS

### 3.1 Identification of differentially expressed genes (DEGs) in heart transplantation (HTx) rejection endomyocardial biopsies (EMBs)

**Figure 1:**
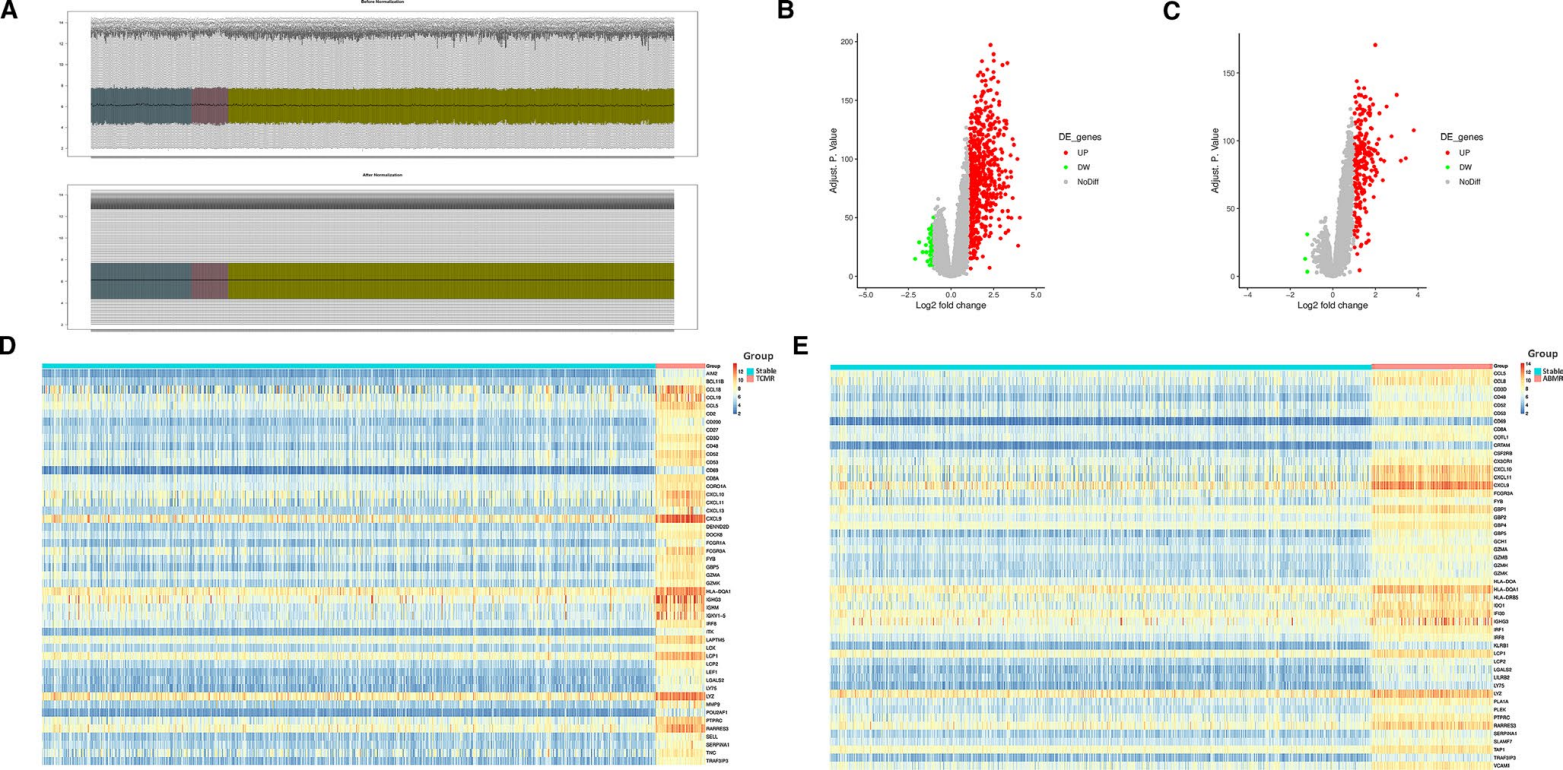
Identification of differentially expressed genes (DEGs) in endomyocardial biopsies (EMBs) from T cell–mediated rejection (TCMR) and antibody‐mediated rejection (ABMR) heart transplantation (HTx) compared with stable HTx. (A) The box plot shows the normalization process of the data. The stable, TCMR and ABMR samples were marked in yellow, pink and blue, respectively. (B) Volcano plots of all DEGs in the TCMR group. (C) Volcano plots of all DEGs in the ABMR group. (D) Heat maps of the top 50 most DEGs in the TCMR group. (E) Heat maps of the top 50 most DEGs in the ABMR group

The normalization process of the data performed by the R LIMMA package is shown in Figure 1A. A total of 18,835 genes were detected in HTx EMBs. Compared with the stable group, 740 and 231 DEGs were identified in the TCMR and ABMR groups, respectively (Table S1). The most differentially expressed gene in each of the TCMR and ABMR groups was C‐X‐C motif chemokine ligand 9 (log_2_ FC = 4.04) and the C‐X‐C motif chemokine ligand 11 (log_2_ FC = 3.80), respectively. The volcano plots for DEGs, TCMR vs stable and ABMR vs stable, are shown in Figure 1A,B, respectively, and the expression levels of the top 50 DEGs in the TCMR and ABMR groups are represented by heat maps in Figure 1C,D, respectively.

### 3.2 Functional enrichment analyses of DEGs associated with HTx rejection reveal roles in the immune response

**Figure 2:**
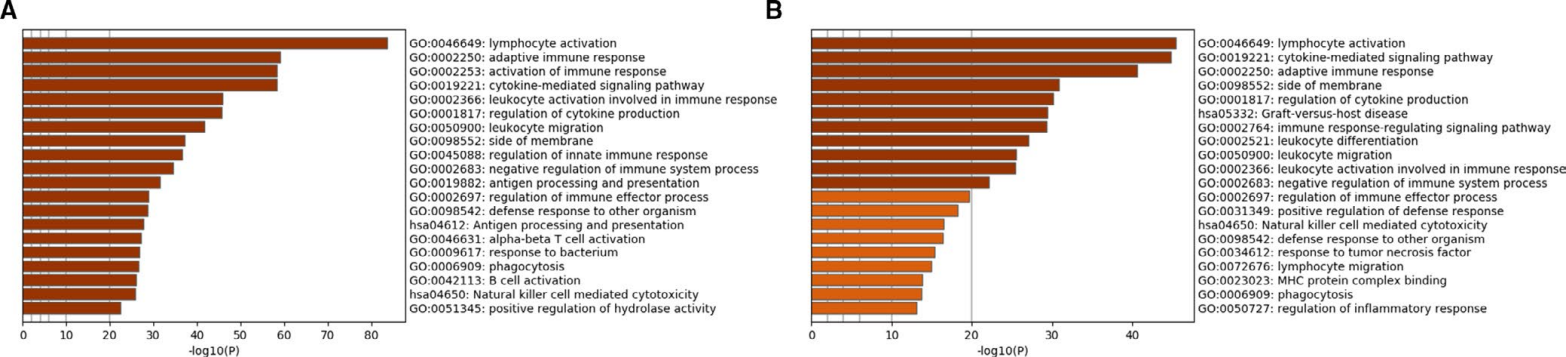
Functional enrichment analysis of DEGs of TCMR (A) and ABMR (B). Top 20 items coloured according to P values

Functional enrichment analyses using the *Metascape* tool revealed that DEGs in both TCMR and ABMR groups were enriched mainly in pathways related to the immune response (Figure 2A,B, respectively), such as ‘lymphocyte activation’ (GO: 0046649), ‘cytokine‐mediated signaling pathway’ (GO: 0019221), ‘adaptive immune response’ (GO: 0002250) and ‘regulation of cytokine production’ (GO: 0001817).

### 3.3 Protein‐protein interaction (PPI) network analysis identified hub genes common to both TCMR and ABMR HTx

**Figure 3:**
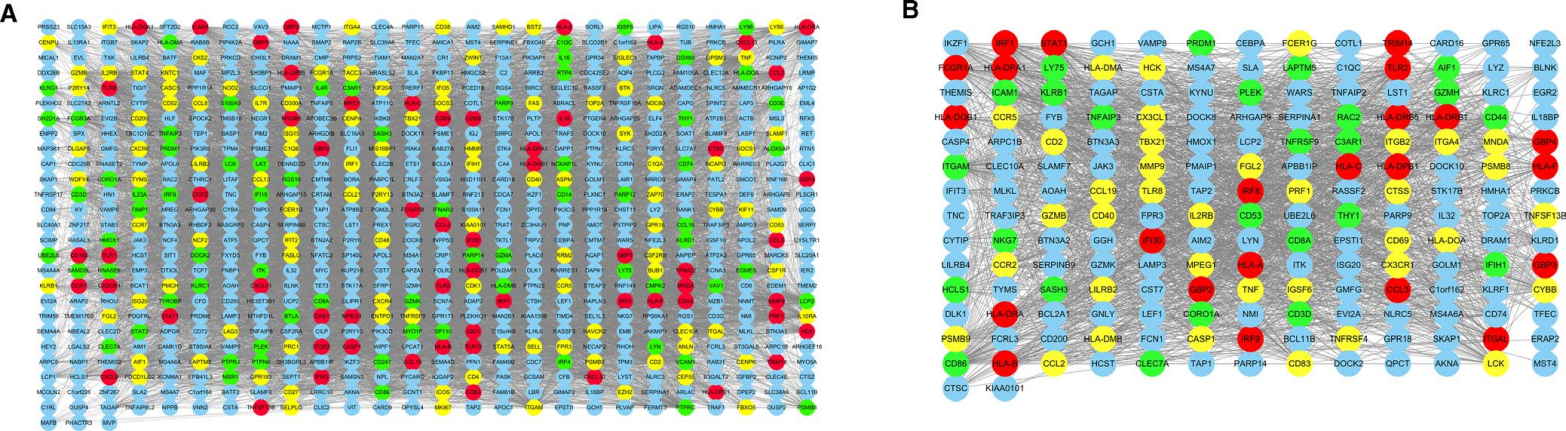
The protein‐protein interaction network of DEGs. (A) TCMR group. (B) ABMR group. Nodes represent DEGs. Red, yellow and green nodes in each group represent top‐ranked modules 1, 2 and 3, respectively

The PPI networks of DEGs in the TCMR and ABMR groups were constructed using the STRING database and visualized using *Cytoscape* software. PPI networks were constructed with 679 nodes and 10 511 edges in the TCMR group (Figure 3A) and 184 nodes and 2146 edges in the ABMR group (Figure 3B). The three top‐ranked modules from each PPI network, TCMR and ABMR groups, respectively, were identified using the MCODE plug‐in (Figure 3). Functional enrichment analyses revealed that DEGs in these modules were mainly associated with immune‐related processes, suggesting that immune dysregulation plays a critical role in HTx rejection (Table 2).

**Table 2:** Functional enrichment analyses of DEGs in top‐ranked modules

Top central module	Term	Description	Count	Log *P* value
TCMR vs Stable				
Module 1				
60 nodes; 876 edges.	GO: 0071346	Cellular response to interferon gamma	35	−59.29
MCODE scores = 29.695	hsa05168	Herpes simplex infection	21	−29.23
	GO: 0001817	Regulation of cytokine production	25	−21.62
Module 2				
107 nodes; 1303 edges.	GO: 0019221	Cytokine‐mediated signalling pathway	36	−26.193
MCODE scores = 24.585	GO: 0 007 159	Leucocyte cell‐cell adhesion	25	−22.715
	GO: 0050900	Leucocyte migration	23	−16.499
Module 3				
71 nodes; 425 edges.	GO: 0042110	T cell activation	27	−27.442
MCODE scores = 12.143	R‐HSA‐1280218	Adaptive immune system	29	−24.60
	GO: 0002253	Activation of immune response	25	−19.814
ABMR vs Stable				
Module 1				
23 nodes; 191 edges.	R‐HSA‐877300	Interferon gamma signalling	20	−46.18
MCODE scores = 17.364	R‐HSA‐909733	Interferon alpha/beta signalling	9	−17.27
	R‐HSA‐1280218	Adaptive immune system	14	−15.34
Module 2				
37 nodes; 272 edges.	GO: 0002694	Regulation of leucocyte activation	18	−19.16
MCODE scores = 15.111	GO: 0019221	Cytokine‐mediated signalling pathway	18	−16.83
	GO: 0001817	Regulation of cytokine production	16	−14.36
Module 3				
25 nodes; 90 edges.	GO: 0042110	T cell activation	12	−14.00
MCODE scores = 7.5	GO: 0050900	Leucocyte migration	8	−7.60
	GO: 0032663	Regulation of interleukin‐2 production	4	−6.55

To identify hub genes, the cytoHubba plug‐in was applied to identify the top 10 DEGs in the PPI network ranked by the MCC method. Four genes, intercellular adhesion molecule 1 (*ICAM1*), CD44 molecule (*CD44*), major histocompatibility complex, class I, A (*HLA‐A*) and major histocompatibility complex, class I, B (*HLA‐B*), were in the top 10 most DEGs in both TCMR and ABMR groups (Table 3) and thus identified as hub genes for HTx rejection. Notably, in the TCMR group, all four hub genes were in Module 1. In the ABMR group, *HLA‐A* and *HLA‐B* were in Module 1, and *ICAM1* and *CD44* were in Module 3.

**Table 3:** Top 10 differentially expressed genes (DEGs) in the TCMR and ABMR groups ranked by MCC method. The four common DEGs *ICAM1, CD44, HLA‐A* and *HLA‐B* were marked in bold font and identified as hub genes for HTx rejection

Group	Rank	Gene Name	Score
TCMR	1	***ICAM1***	5.27E + 27
	2	***CD44***	5.18E + 27
	3	*VCAM1*	4.31E + 27
	4	*FCGR1A*	3.29E + 27
	5	*IRF4*	3.29E + 27
	6	*IRF1*	3.29E + 27
	7	*IRF9*	3.29E + 27
	8	***HLA‐A***	3.29E + 27
	9	*HLA‐E*	3.29E + 27
	9	***HLA‐B***	3.29E + 27
ABMR	1	***ICAM1***	1.28E + 17
	2	***CD44***	1.28E + 17
	3	*HLA‐DRB1*	1.28E + 17
	4	***HLA‐A***	1.28E + 17
	5	*HLA‐DQB1*	1.28E + 17
	6	***HLA‐B***	1.28E + 17
	7	*HLA‐DRA*	1.28E + 17
	8	*HLA‐DPA1*	1.28E + 17
	9	*HLA‐C*	1.28E + 17
	10	*HLA‐DPB1*	1.28E + 17

### 3.4 Immune cell infiltration is altered in TCMR and ABMR samples

**Figure 4:**
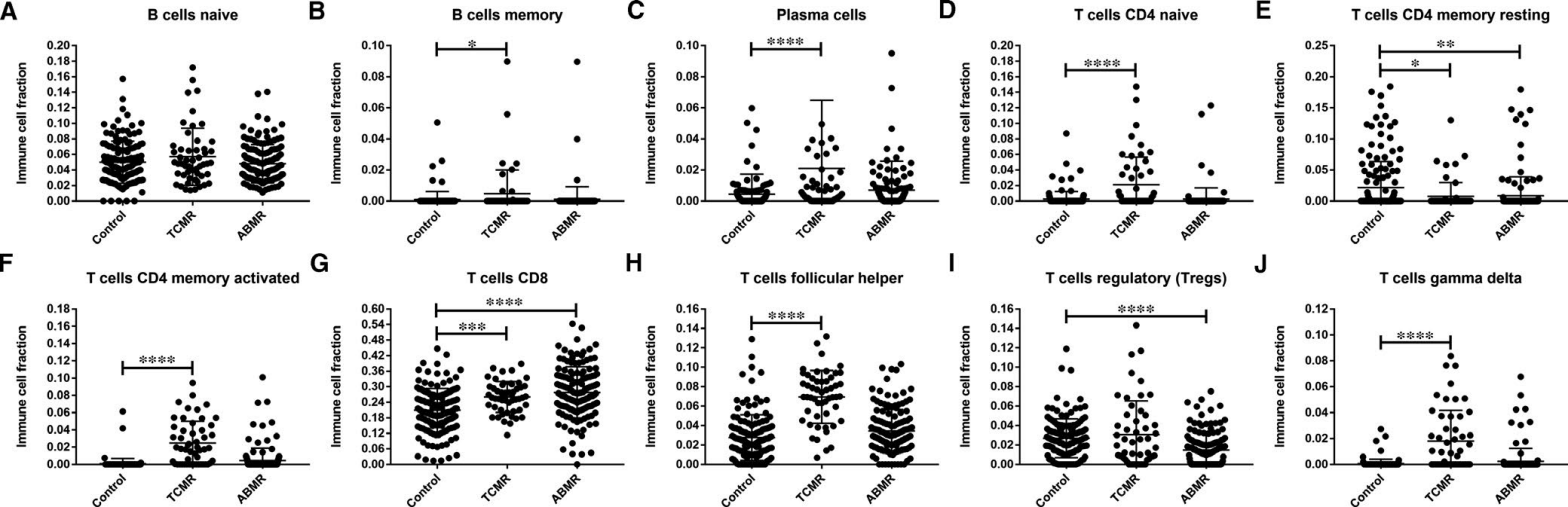
Cells of the adaptive immune system in stable, TCMR and ABMR HTx EMB samples. (A) Naive B cells, (B) Memory B cells, (C) Plasma cells, (D) CD4^+^ naive T cells, (E) Resting memory CD4^+^ T cells, (F) Activated memory CD4^+^ T cells, (G) CD8^+^ T cells, (H) Follicular helper T cells, (I) Tregs and (J) γδ T cells. *P < .05; **P < .01; ***P < .001; ****P < .0001

**Figure 5:**
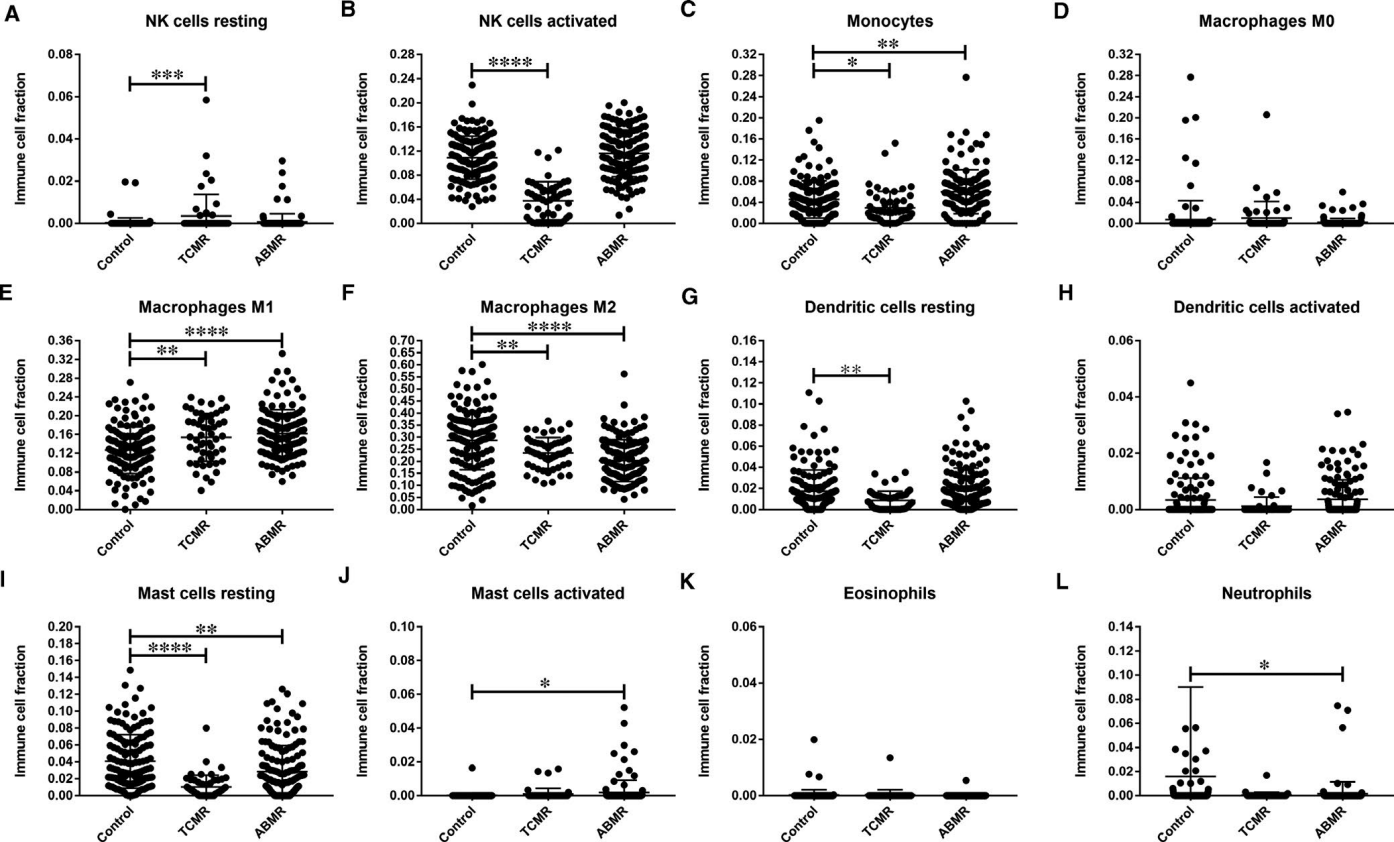
Cells of the innate immune system in stable, TCMR and ABMR HTx EMB samples. (A) Resting NK cells, (B) Activated NK cells, (C) Monocytes, (D) M0 macrophages, (E) M1 macrophages, (F) M2 macrophages, (G) Resting dendritic cells, (H) Activated dendritic cells, (I) Resting mast cells, (J) Activated mast cells, (K) Eosinophils and (L) Neutrophils. *P < .05; **P < .01; ***P < .001; ****P < .0001

According to the CIBERSORT algorithm, 143 stable, 52 TCMR and 142 ABMR HTx EMBs that matched the requirements of CIBERSORT *P* < .05 were identified after filtering of data (Table S2). Differences in adaptive and innate immune cell populations between TCMR, ABMR and stable HTx samples were investigated (Figures 4 and 5, respectively). Among adaptive immune cells, higher proportions of memory B cells, plasma cells, naïve CD4^+^ T cells, activated memory CD4^+^ T cells, CD8^+^ T cells, follicular helper T cells and γδ T cells were detected in TCMR samples compared to stable samples, along with lower proportions of resting memory CD4^+^ T cells (Figure 4B‐H,J). In ABMR samples compared to stable samples, greater proportions of CD8^+^ T cells were detected, along with lower proportions of resting memory CD4^+^ T cells and Tregs (Figure 4E,G,I). Among innate immune cells, higher proportions of resting natural killer (NK) cells and M1 macrophages were detected in TCMR samples compared to other samples, along with lower proportions of activated NK cells, monocytes, M2 macrophages and resting mast cells (Figure 5A‐C,E‐G,I). In addition, higher proportions of monocytes, M1 macrophages and activated mast cells were detected in ABMR samples compared to stable samples, along with lower proportions of M2 macrophages, resting mast cells and neutrophils (Figure 5C,E,F,I,J,L).

### 3.5 Significant correlations between hub gene levels and immune cell proportions

**Figure 6:**
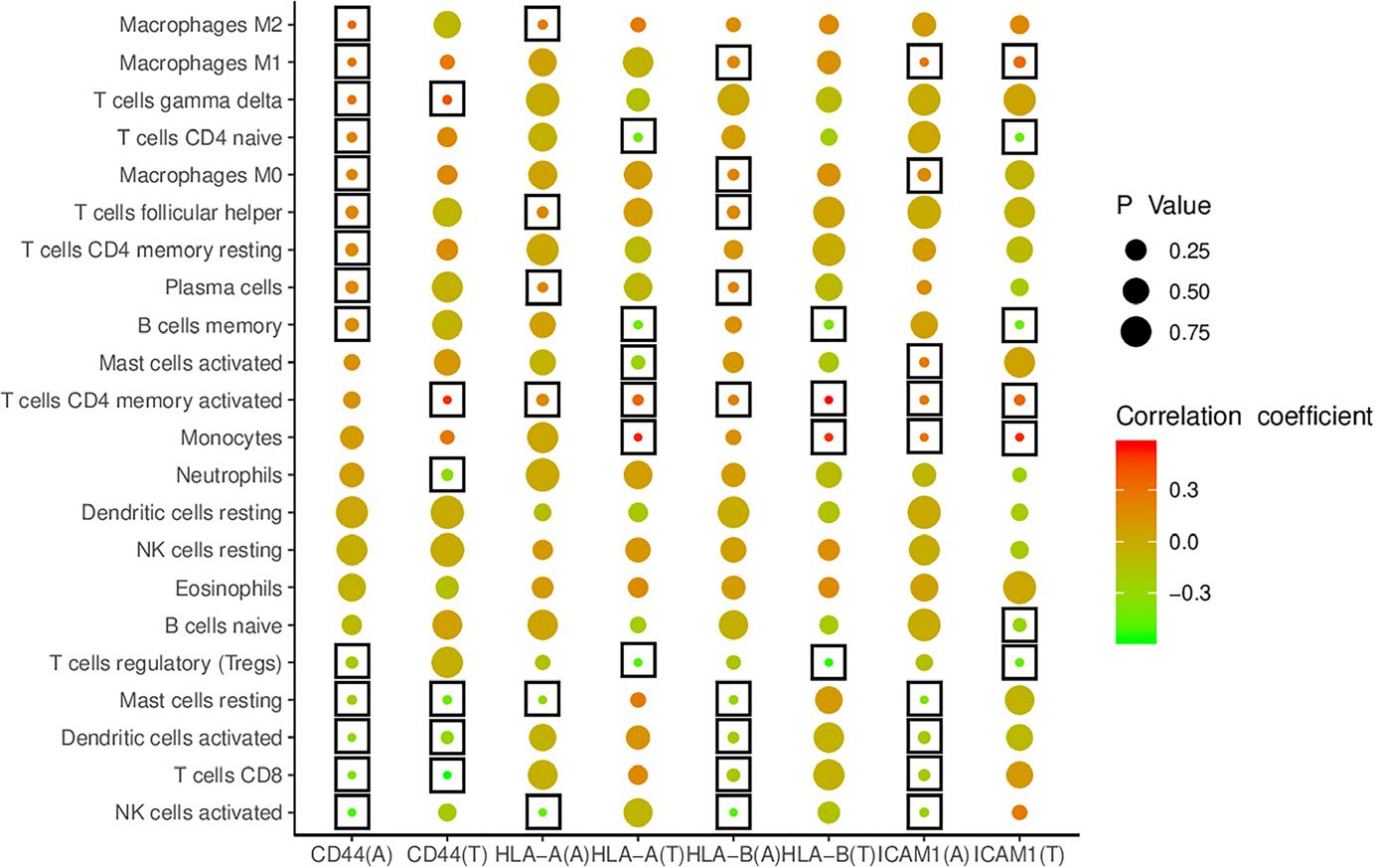
Correlation indices between hub gene expression levels and immune cell infiltration levels in HTx samples. A: ABMR; T: TCMR. Significant correlations (*P* < .05) between hub gene levels and representative proportions of immune cells are marked with boxes

Correlation analysis revealed relationships between hub gene levels and immune cell proportions in TCMR and ABMR groups (Figure 6). In the TCMR group, *ICAM1* expression significantly correlated with 7 immune cell types, *CD44* expression significantly correlated with 6 immune cell types, *HLA‐A* expression significantly correlated with 6 immune cell types, and *HLA‐B* expression significantly correlated with 4 immune cell types. In the ABMR group, *ICAM1* expression significantly correlated with 9 immune cell types, *CD44* expression significantly correlated with 14 immune cell types, *HLA‐A* expression significantly correlated with 6 immune cell types, and *HLA‐B* expression significantly correlated with 9 immune cell types. These hub gene/immune cell correlations indicate that *ICAM1*, *CD44*, *HLA‐A* and *HLA‐B* may participate in the immune response during cardiac allograft rejection by affecting the composition of the immune cell infiltration.

### 3.6 Drug‐gene interaction analysis predicts FDA‐approved drugs with potential to target hub genes

Based on DGIdb prediction, a total of 13 drug‐gene interactions were identified, including 4 hub genes and 12 FDA‐approved drugs (Figure S1). *HLA‐A* can be targeted by 6 drugs (thalidomide, oxcarbazepine, pazopanib, phenytoin, abacavir and carbamazepine), *CD44* can be targeted by 4 drugs (gentamicin, mycophenolic acid, interferon γ‐1b and mometasone furoate), *ICAM1* can be targeted by 2 drugs (lifitegrast and natalizumab), and *HLA‐B* can be targeted by one drug (carbamazepine).

### 3.7 Validation of hub genes

**Figure 7:**
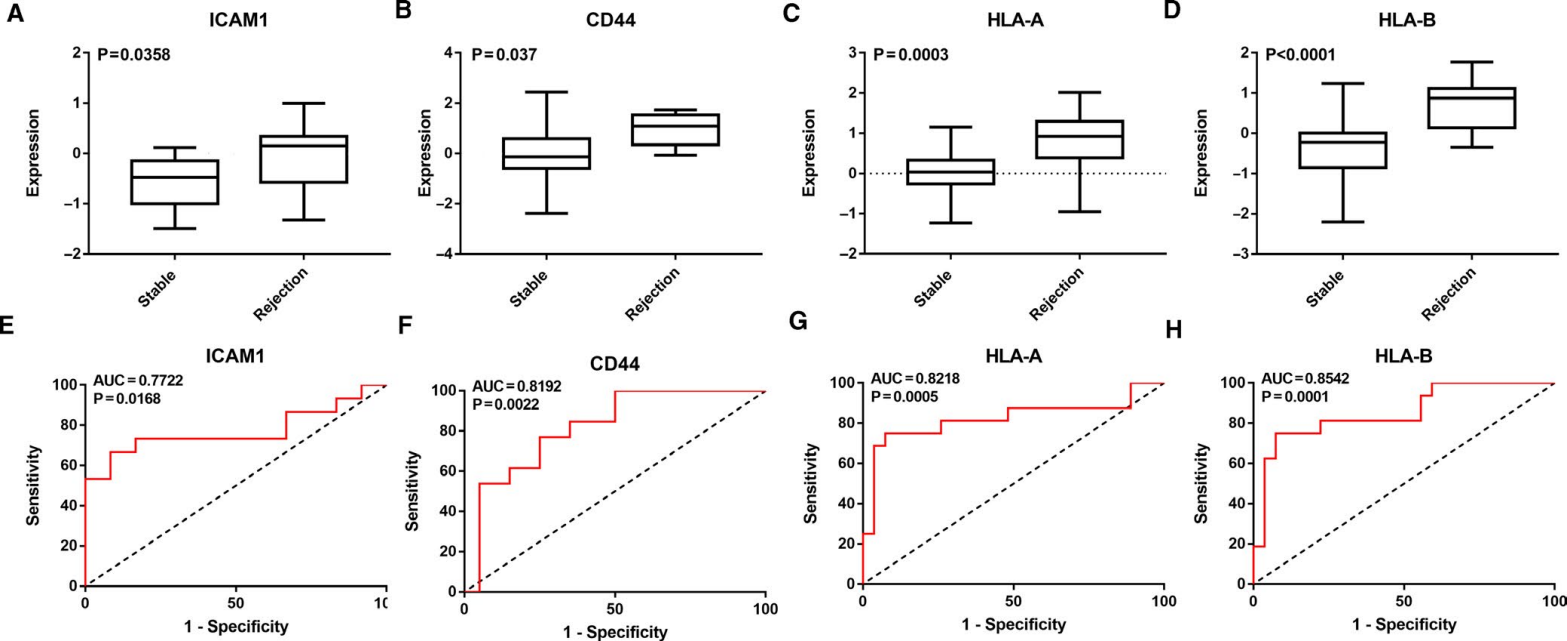
Validation of hub gene expression level and diagnostic value of using the GSE2596 data set. The expression of (A) ICAM1, (B) CD44, (C) HLA‐A and (D) HLA‐B in rejection HTx and stable HTx samples. The *y*‐axis represents the relative expression of hub genes. *P* value <.05 indicates a statistically significant difference between the hub gene expression in rejection samples compared with the stable samples. The receiver operating characteristic (ROC) curves were used to evaluate the diagnostic value of (E) ICAM1, (F) CD44, (G) HLA‐A and (H) HLA‐B. The *x*‐axis shows 1‐specificity, and the *y*‐axis shows sensitivity. AUC: area under the ROC curve

The external GSE2596 data set was used to validate the expression levels and diagnostic value of the hub genes. All 4 hub genes (*ICAM1*, *CD44*, *HLA‐A* and *HLA‐B*) are expressed at significantly higher levels in rejection HTx samples than in stable HTx samples (Figure 7A‐D). To assess the diagnostic value of hub genes, ROC curves were also generated. The expression of all 4 hub genes was significantly associated with a diagnosis of HTx rejection (0.7 < AUC <1, *P* < .05) (Figure 7E‐H).

## 4 DISCUSSION

T cell‒mediated rejection and ABMR have emerged as major risk factors for limiting heart allograft survival.^19^, ^20^, ^21^ However, the differences in gene expression and immune status of rejection and stable heart graft samples are still unknown. Bioinformatics analysis identified a total of 740 and 231 DEGs in the TCMR and ABMR groups, respectively. Functional enrichment analysis revealed that DEGs in the TCMR and ABMR groups belong mainly to processes associated with the immune response, indicative of the abnormal immune regulation that occurs during cardiac allograft rejection.^22^

In the PPI network construction, the top‐ranked DEGs were selected based on the MCC method, and four genes, *ICAM1*, *CD44*, *HLA‐A* and *HLA‐B*, were identified as hub genes. All four genes were overexpressed in both TCMR and ABMR groups. ICAM‐1 is an adhesion molecule that contributes to transplant rejection by inducing the transendothelial migration and infiltration of leucocytes into graft tissues.^23^ In animal models of HTx, ICAM‐1 expression is critical for both acute and chronic cardiac transplant rejection,^24^, ^25^, ^26^, ^27^ and blocking the ICAM‐1/lymphocyte function‐associated antigen 1 (LFA‐1) pathway with ICAM‐1‐ and LFA‐1‐specific monoclonal antibodies (mAbs) can promote heart graft tolerance and prolong graft survival.^28^, ^29^, ^30^ CD44 is another cell‐surface adhesion molecule that activates T cell responses following HTx. Inhibition of CD44 with mAbs can suppress accelerated heart allograft rejection in mice.^31^, ^32^ HLA‐A and HLA‐B are two alleles of major histocompatibility complex (MHC) class I, whose compatibility is critical for organ transplant survival.^33^ However, in several studies, no benefits of HLA‐A/B matching were found on heart graft survival, rejection or infection,^34^, ^35^, ^36^ and how HLA‐A/B overexpression affects heart graft rejection is unclear.

In a previous study, Lionetti et al^37^ performed proteomic analysis of different myocardial regions of end‐stage failing hearts of patients eligible for HTx, which reveals the mechanisms of heart failure occurrence at the protein level. Since heart failure is one of the main causes of rejection following HTx, we should also focus on the relationships between the four hub genes *ICAM1*, *CD44*, *HLA‐A* and *HLA‐B* and heart failure: Intramyocardial endothelial ICAM1 was found to promote cardiac inflammation and pathological cardiac remodelling by promoting T cell recruitment in the left ventricle, which contributes to cardiac fibrosis and dysfunction and heart failure.^38^ CD44 is also crucial in the development of cardiac remodelling and myocardial fibrosis, which accelerates the progression to heart failure.^39^ In addition, HLA‐A and HLA‐B also play potential roles in heart failure.^40^ These findings suggest that it is worth investigating the exact role of these hub genes in heart failure following HTx and whether the expression of these hub genes in different myocardial regions has different patterns to affect the development of heart failure following HTx.

The CIBERSORT algorithm defined the immune cell infiltration characteristics in the HTx samples and revealed abnormal adaptive and innate immunity in the TCMR and ABMR groups. Significant differences in the proportions of several types of immune cells were found in our study, which were consistent with other studies: (a) The abundance of B cells, including CD20^+^CD27^+^ memory B cells and CD20^‐^CD138^+^ plasma cells, in heart graft infiltrates is closely associated with cardiac allograft vasculopathy.^41^ (b) Activated CD4^+^/CD8^+^ T cell responses play a critical role in heart graft rejection.^19^, ^42^, ^43^ (c) Activated pro‐inflammatory follicular helper T cells help to generate donor‐specific antibody (DSA) responses after organ transplantation, which is an attractive target for improving the effects of immunosuppressive drugs.^44^, ^45^ (d) Treg therapy is a novel tool for delaying graft rejection following solid organ transplantation.^46^ Expanding the frequency of anti‐inflammatory FoxP3^+^ Tregs prevents heart graft rejection and prolongs graft survival in mice.^47^, ^48^, ^49^ (e) Activated γδ T cells promote heart graft rejection in mice by inducing IL‐17 production, which can be reversed by depletion of γδ T cells.^50^, ^51^ (6) NK cells can induce allograft tolerance.^52^, ^53^ In mice and rat model of HTx, impairment of NK cell response results in acceleration of rejection and prevents the establishment of graft acceptance.^54^, ^55^ (7) Activated macrophages are essential in heart allograft rejection, depletion of which can promote graft acceptance in mice.^56^, ^57^

Analysis in this study revealed that the expression levels of hub genes in HTx rejection samples was significantly correlated with several altered proportions of immune cells, which may partly explain how these hub genes contribute to HTx rejection through the regulation of immune responses, such as (a) *ICAM1* expression was positively correlated with the proportions of activated memory CD4^+^ T cells and M1 macrophages in TCMR/ABMR samples. (b) *CD44* expression was positively correlated with the fraction of γδ T cells in TCMR/ABMR samples. (c) *HLA‐A* and HLA*‐B* expression was positively correlated with the proportion of activated memory CD4^+^ T cells in TCMR/ABMR samples.

Potential drug‐gene interactions were further investigated, and 12 FDA‐approved drugs were predicted to target hub genes. Of note, immunosuppressive functions of some drugs have been found. Thalidomide as an immunosuppressant can effectively prevent HTx rejection in animal models.^58^, ^59^ Mycophenolic acid, another immunosuppressant, has been widely used for liver, renal and heart transplantation.^60^, ^61^, ^62^ Lifitegrast is a novel integrin antagonist that blocks the binding of ICAM‐1 to LFA‐1, thus inhibiting T cell–mediated inflammation.^63^ Natalizumab, an integrin α4β1 (VLA‐4) antagonist that interferes with leucocyte trafficking by blocking the interaction between VLA‐4 and vascular cell adhesion molecule 1 (VCAM‐1), has been used in the therapy of inflammatory bowel disease and multiple sclerosis.^64^, ^65^ However, in addition to the above drugs, the immunosuppressive effects of other identified drugs have not been reported, and whether these 12 drugs are appropriate for immunosuppression in HTx needs further experimental verification.

Finally, using the external GSE2596 data set, the high expression levels and diagnostic values of the hub genes were validated, indicating their potential for the diagnosis and treatment of HTx rejection.

The clinical and practical implications of results in the present study are as follows: (a) The identified hub genes may serve as potential therapeutic targets for HTx rejection, which has been confirmed in some previous studies.^24^, ^25^, ^26^, ^27^, ^28^, ^29^, ^30^, ^31^, ^32^ (b) The identified hub genes may have potential diagnostic value for the diagnosis of HTx rejection. (c) The abnormal immune status in rejection HTx samples and the close relationships between hub gene levels and immune infiltrates revealed in this study provide a reference for future studies to investigate molecular immunopathological mechanisms of HTx rejection that may be useful for developing relevant treatment strategies. For example, whether suppressing the expression of these hub genes can protect heart transplants by regulating transplant immunity.

In summary, DEGs between rejection and stable EMBs were screened using bioinformatics analysis. According to the PPI network of DEGs, *ICAM1*, *CD44*, *HLA‐A* and *HLA‐B* were identified as hub genes. Differences in the composition of immune cell infiltration between rejection and stable HTx EMBs were revealed: high proportions of pro‐inflammatory immune cells such as CD4^+^/CD8^+^ T cells and M1 macrophages, and low proportions of anti‐inflammatory immune cells such as Tregs and M2 macrophages were found in rejection EMBs. In addition, close relationships between hub gene expression levels and the immune cell infiltration composition were also revealed, such as the positive correlation between *ICAM1* expression and activated memory CD4^+^ T cells and M1 macrophages; the positive correlation between *CD44* expression and γδ T cells; the positive correlation between *HLA‐A* and *HLA‐B* expression and activated memory CD4^+^ T cells. Moreover, 12 FDA‐approved drugs that potentially target the hub genes were identified. Finally, hub genes were validated using the GSE2596 data set.

Inevitably, the present study had several limitations. Some key information on clinical and laboratory characteristics of the patients in the present study such as comorbidities, medications, cardiac function and haemodynamics, and laboratory biomarkers were unavailable from the results of NCT02670408. What's more, some patient/biopsy characteristics may potentially affect on our results, which needs further exploration. In addition, the present study lacked further experimental verification as a solid foundation. However, the results of the current study provide a strong basis on which to develop future studies to obtain a more detailed understanding of the molecular and immune mechanisms underlying HTx rejection, and thus improving strategies to diagnose and prevent HTx rejection.

## CONFLICT OF INTEREST

The authors declare that they have no known competing financial interests or personal relationships that could have appeared to influence the work reported in this paper.

## AUTHOR CONTRIBUTION

**Mengxi Xiu:** Data curation (equal); Formal analysis (equal); Resources (equal); Supervision (equal); Validation (equal); Visualization (equal); Writing‐original draft (equal). **yuanmeng Liu:** Software (equal); Validation (equal); Writing‐review & editing (equal). **wenjun Wang:** Formal analysis (equal); Funding acquisition (equal); Project administration (equal).

## Supporting information

Figure S1

Table S1

Table S2

## Data Availability

The datasests generated and/or analyzed during the current study are available from the Gene Expression Omnibus GSE124897 and GSE2596.
