# Supplementary figures and images for: Investigation of hub genes and immune status in heart transplant rejection using endomyocardial biopsies
Source: J Cell Mol Med. 2020 Nov 23;25(2):763–73. doi: 10.1111/jcmm.16127 (PMC7812257; doi:10.1111/jcmm.16127)

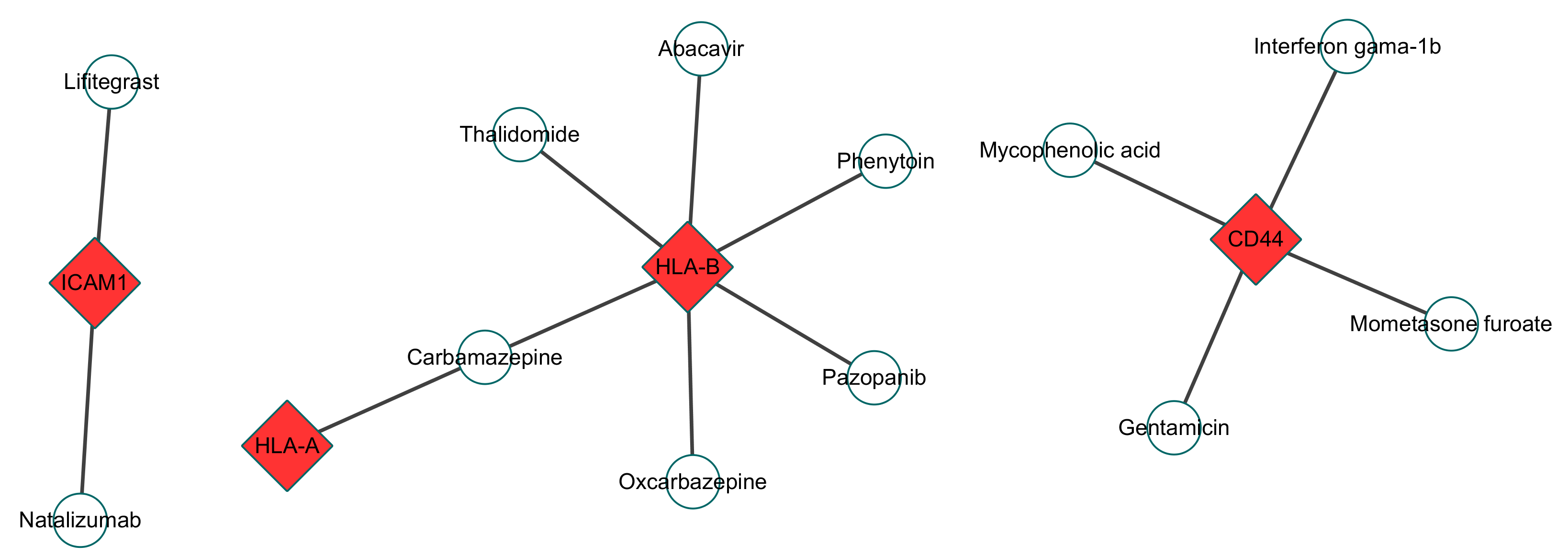

Supplement: Supplementary file 1 — Figure S1 [file JCMM-25-763-s001.tif]
